# Examining Parental Monitoring and Deviant Peers as Pathways to Sexual Risk Behaviors among Tehran (IR Iran) Adolescents

**DOI:** 10.5812/ijhrba.14815

**Published:** 2013-09-20

**Authors:** Dexter R Voisin

**Affiliations:** 1 School of Social Service Administration, University of Chicago, Chicago, USA

**Keywords:** Unsafe Sex, Parenting, Peer Group, Adolescent

*Dear Editor,*

The article by Ahmadi et al. documented that boys affiliated more with deviant peers, experienced higher rates of riskier sex and lower levels of parental monitoring compared to girls among a sample of 1266 Tehran students. Additionally, the authors reported direct relationships between parental monitoring and sexual risk behaviors. Finally, results provided evidence that negative peer norms mediated the relationship between parental monitoring and sexual risk behaviors (1). Taken together, these findings make important contributions to the extant literature, corroborating earlier findings (2, 3) and articulating important gender differences that warrant further study. The literature on youth development often supports multiple perspectives on the trajectory of youth problem behaviors. One common perspective asserts that the influence of parental figures diminishes while that of peers increases. Another posits that the significance of negative peer influences only increases to the extent that strong parental monitoring functions are absent. The findings of this study reinforce these two perspectives and support their accuracy, at least among this population.

However, not unlike many scientific studies, these findings seem to present more questions than definitive answers. First, there were no clear temporal stems for major study variables (i.e. parental monitoring, negative peer norms, or sexual risk behaviors). Therefore we do not know the temporal ordering of these variables and it is likely that many of the significant relationships in this study are bidirectional. For instance, it is plausible that engaging in risky sexual behaviors may draw youth into cohorts of peers who endorse drug and alcohol use or vice-versa. More importantly, cross-sectional studies like this one are limited in their ability to tease out temporal orderings or infer causal inferences. Given the methodological limitations of cross-sectional approaches, I find it highly unorthodox when the language of ‘prediction’ is associated with findings based on this method. However, notwithstanding these limitations, cross-sectional findings are highly valuable in establishing the existence of important relationships prior to developing costly longitudinal studies, which might then clarify temporal associations.

As the authors noted, there are few studies that have examined the dynamics of sexual risk-taking, peer influences and parental monitoring in Tehran. Consequently, the significance of this study is timely. However, it remains unclear when this study was conducted as dates are not included. The authors mention that “4.5% of [teens] reported distress in the structure of their family;” although it is unclear what this distress refers to, such language suggests that important additional variables were collected in this survey. Using a broader ecological perspective, it would be intriguing to explore how community-level factors (e.g. community violence exposure or distress, gender roles and expectations and/or other cultural constructs) might impact parental monitoring and the pathways to risk explored in this study. In addition, given that the results of separate structural equation models were not reported in this study, nor was gender treated as a covariate, it is unclear whether to pathways to risk noted in this study were in fact gendered. Increasingly, research is documenting that although adolescent males and females may engage in similar types of risk behaviors, though at times to varying degrees, the mechanisms to such behaviors are often gendered (4). Based on these study findings, and given the unique gender and cultural scripts present in Tehran, there is still much to learn about the trajectories of youth problem behaviors in this region.
